# Editorial: Whole Genome Sequencing for rare diseases

**DOI:** 10.3389/fmed.2023.1267930

**Published:** 2023-09-19

**Authors:** Chiara Di Resta, Valeria D'Argenio

**Affiliations:** ^1^Faculty of Medicine, Vita-Salute San Raffaele University, Milan, Italy; ^2^Genomic Unit for the Diagnosis of Human Pathologies, IRCCS San Raffaele Scientific Institute, Milan, Italy; ^3^Department of Human Sciences and Quality of Life Promotion, San Raffaele Open University, Rome, Italy; ^4^CEINGE-Biotecnologie Avanzate Franco Salvatore, Naples, Italy

**Keywords:** rare diseases, Whole Genome Sequencing, diagnosis, treatment, pathogenesis

According to the European Union definition, a disease is considered rare when it affects less than 1 in 2,000 citizens (1). To date, about 10,000 rare diseases (RDs) are known, afflicting at least 4–6% of the worldwide population. RDs are featured in a wide range of clinical phenotypes, also among patients suffering from the same disorder (2). It is estimated that 80% of RDs have a genetic etiology and 70% have childhood onset (3).

The identification of the causative variant(s) and the mode of inheritance are crucial steps in the definition of an accurate diagnosis and lead to better disease management, avoiding unnecessary treatments (4). However, even when the etiology of the disease is known, overlapping phenotypes and genetic testing limited to disease-targeted genes panels severely weaken the chances of the diagnosis (5).

The RDs' diagnostic delay varies from months to decades, depending on the patient's phenotype, age, and available resources. It is estimated that the average time for an accurate RD diagnosis is about 5 years and, in some cases, it can take over a decade (3). This delay is defined as diagnostic odyssey, a period during which, despite extensive and expensive workups at several institutions, patients often remain undiagnosed or even misdiagnosed, with consequent delays in getting effective care and further emotional distress for patients and their family members (5).

One of the main challenges in the diagnosis of RDs is their rarity. Indeed, many healthcare professionals may never encounter a patient with a particular RD during their entire career, making it difficult to recognize symptoms and make an accurate diagnosis (6, 7). Therefore the lack of knowledge and awareness regarding RDs are sometimes the main factors limiting the primary care of affected patients.

In this scenario, the advent of next-generation sequencing has had a dramatic effect on the step forward in the identification of the molecular bases, the pathogenesis and the clinical prediction targets for personalized treatment, improving especially the diagnostic yield (8, 9).

In particular, Whole Genome Sequencing (WGS) offers the highest likelihood of identifying the causative mutations of an uncharacterized genetic condition, supplanting the older sequencing technologies in this field (10).

This Research Topic encompasses these aspects, with an exemplificative article collection on the huge potential of WGS.

In particular, Dai et al. describe the case of a patient affected by a very rare dermatological disorder, the Mal de Meleda (MDM), that is often misdiagnosed due to its phenotypic overlapping with other forms of palmoplantar keratoderma. Moreover, many dermatologists are not familiar with this rare disease, leading to a delay in the appropriate treatments. In the described case, the causative mutation was identified by WGS, thus allowing a correct diagnosis and the definition of the most effective treatment (Dai et al.).

A similar example is described by Xiong et al. who, exploiting WGS, reached the clinical diagnosis of a case with suspected familial pseudohyperkalemia (FP) caused by red blood cell membrane defects. FP is a benign condition for which an inappropriate potassium-lowering therapy might lead to detrimental outcomes, highlighting the importance of defining the right clinical diagnosis (Xiong et al.).

On this crucial theme, Chu et al. gave an important contribution, showing the application of WGS also in prenatal diagnosis, particularly in the identification of pathogenic copy number variations (CNVs). The Authors reported three prenatal diagnosis pedigrees in which they demonstrated that CNV-seq could be a useful approach for the definition of chromosomal breakpoints related to the 5p deletion syndrome, thus providing helpful genetic guidance to at-risk families and shortening the diagnostic process for timely follow-up interventions (Chu et al.).

Furthermore, WGS has been used to identify novel genetic mutations in rare conditions, whose molecular background is still unknown, as described by Chen et al., and allowed to highlight a significant association between a specific HLA genotype and severe hemophilia A, as reported by Lessard et al.. These two articles are examples of contributions demonstrating the important role of WGS in improving both the diagnostic process and the choice of the most effective therapy.

As previously described, the clinical overlapping among different RDs is one of the main factors hampering their diagnosis. The identification of the causative molecular background in patients with a spectrum of anomalies and phenotypic variability could be crucial to better define the therapeutic strategy and the personalized clinical follow-up. In this context, Garza Flores et al. described two unrelated patients with *FOXC1* haploinsufficiency, related to Axenfeld-Rieger syndrome and skeletal abnormalities, presenting a spectrum of clinical signs overlapping with the ultrarare De Hauwere syndrome (Garza Flores et al.). The Authors showed how WGS may be a very useful tool for differential diagnosis in cases similar to those described, featured by phenotypic overlapping among several RDs.

Finally, considering the big amount of generated genomic data, WGS could be useful also to study the association between a specific genetic condition, such as Prader-Willy Syndrome (PWS), and cancer predisposition. Indeed, even with some limitations due to the number of analyzed patients and the short follow-up times,  Maya-González et al. described this WGS application to clarify the lifetime cancer risk in PWS patients (11).

Taken together, the studies published within this Research Topic underline the potentialities of WGS in improving the management of RDs patients, since it can support not only the definition of the precise diagnosis but also the identification of the pathogenic molecular targets and the choice of the most proper therapeutic strategy (Figure 1).

**Figure 1:**
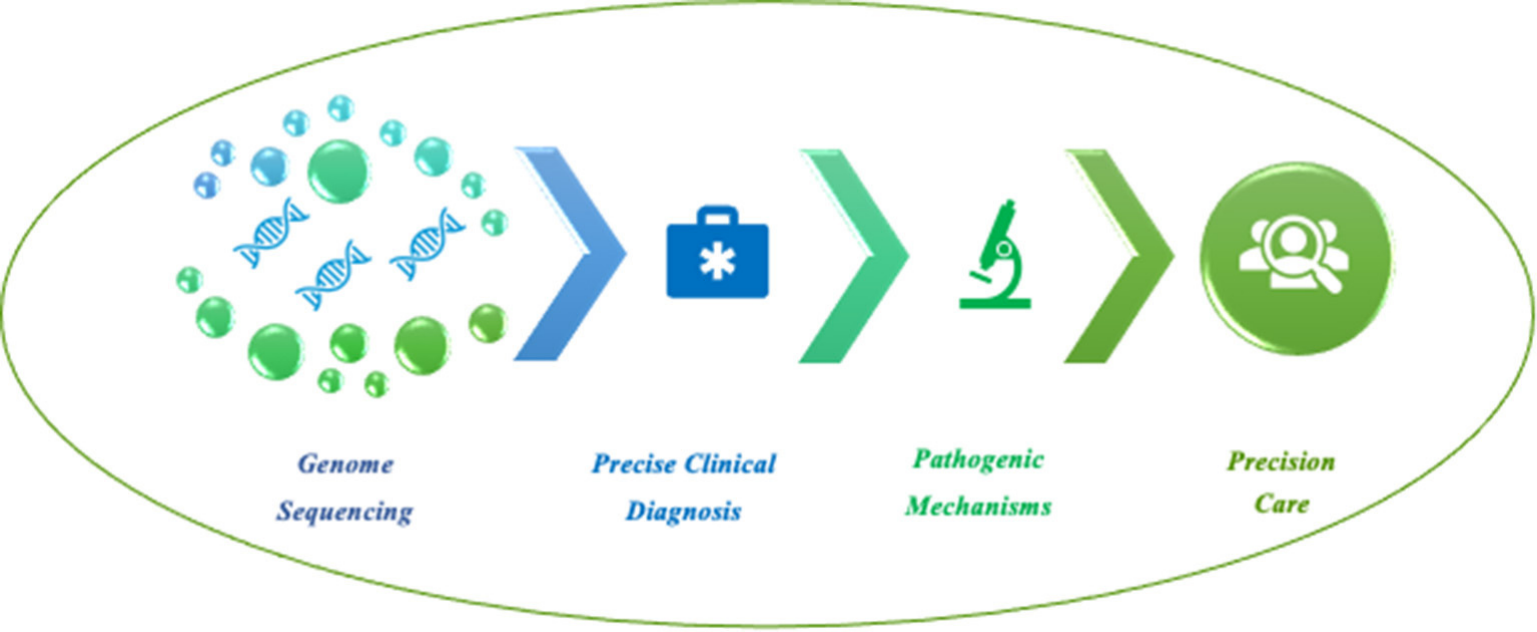
The potential impact of the application of Whole Genome Sequencing in ameliorating the clinical management of rare disorders.

On the other hand, it is necessary to consider that, as significant as the advances are in this field, there are still challenges to be addressed, including the physicians' awareness of RDs and the prioritization and interpretation of such huge amounts of genomic data, to drive the current medicine toward precision medicine (12). For that reason, it is essential to move forward by collaborating and sharing data among multiple RDs referral centers.

## Author contributions

CD: Writing—original draft, Writing—review and editing, Conceptualization, Supervision. VD'A: Writing—original draft, Writing—review and editing, Conceptualization, Supervision.
